# Cinobufotalin inhibits proliferation, migration and invasion in hepatocellular carcinoma by triggering NOX4/NLRP3/GSDMD-dependent pyroptosis

**DOI:** 10.3389/fonc.2024.1438306

**Published:** 2024-10-16

**Authors:** Chen Liu, Jianmin Wu, Zhiwen Li, Xuanyu Huang, Xianhe Xie, Yun Huang

**Affiliations:** ^1^ Department of Oncology, Molecular Oncology Research Institute, The First Affiliated Hospital of Fujian Medical University, Fuzhou, China; ^2^ Department of Oncology, National Regional Medical Center, Binhai Campus of The First Affiliated Hospital, Fujian Medical University, Fuzhou, China; ^3^ Fujian Key Laboratory of Precision Medicine for Cancer, The First Affiliated Hospital of Fujian Medical University, Fuzhou, China; ^4^ Department of Geriatrics, The First Affiliated Hospital of Fujian Medical University, Fuzhou, China; ^5^ Clinical Research Center for Geriatric Hypertension Disease of Fujian Province, The First Affiliated Hospital of Fujian Medical University, Fuzhou, China; ^6^ Department of Neurology, The First Affiliated Hospital of Fujian Medical University, Fuzhou, China; ^7^ Department of Neuroscience, IRCCS - Istituto di Ricerche Farmacologiche Mario Negri, Milan, Italy; ^8^ Department of Medicine, Section of General Pathology, University of Verona, Verona, Italy

**Keywords:** HCC, Cinobufotalin, pyroptosis, NOX4, ROS, NLRP3

## Abstract

**Introduction:**

Pyroptosis is an inflammatory form of programmed cell death that plays a significant role in tumorigenesis. Cinobufotalin (CB), a bufadienolide extracted from toad venom, is associated with antitumor effects in various cancers, including liver cancer. However, the role of CB in pyroptosis and its underlying mechanisms have not been well characterized.

**Methods:**

MTT, Colony formation, EdU, Wound healing and Transwell migration and invasion assays were applied to determine the effects of CB on the proliferation, migration, and invasion ability of hepatocellular carcinoma (HCC) cells *in vitro*. The subcutaneous xenograft mouse model and pulmonary metastasis model were used to evaluate the effect of CB on HCC cells *in vivo*. PCR, western blot, immunohistochemistry, immunofluorescence, and ELISA were used to verify the expression of proliferation, migration, pyroptosis, and inflammation related molecules after CB treatment. Using si-RNA and inhibitors to interfere with NOX4 and HLRP3 expression to validate the key signaling pathways of pyroptosis induced by CB treatment.

**Results:**

*In vivo* experiments using nude mice with xenografted HCC cells and *in vitro* experiments with HCC cell lines demonstrated that CB treatment significantly inhibited the proliferation, migration, and invasiveness of HCC cells. CB treatment also showed dose-dependent activation of the NLRP3 inflammasome complex in HCC cells, leading to gasdermin D-induced pyroptosis. However, these effects were abrogated via the pretreatment of HCC cells with VX-765, a caspase-1 inhibitor. Additionally, CB increased the production of reactive oxygen species (ROS) and H₂O₂, along with upregulating NOX4 protein expression in HCC cells. Conversely, NOX4 silencing or pretreatment with VAS2870 (an NOX4 inhibitor) or NAC (an ROS scavenger) suppressed the activation of the NLRP3 inflammasome complex and pyroptosis in CB-treated HCC cells.

**Discussion:**

Our study demonstrated that CB suppressed the proliferation, migration, and invasiveness of HCC cells by inducing pyroptosis through the activation of the NOX4/NLRP3/GSDMD signaling pathway. Therefore, our results suggest that CB is a promising therapeutic agent for HCC.

## 1 Introduction

Hepatocellular carcinoma (HCC), the most common form of primary liver cancer, is a leading cause of cancer-related deaths worldwide (1, 2). HCC is marked by high incidence, rapid progression, frequent recurrence, and low rates of early diagnosis and survival (3). Various treatment options, including surgery, radiotherapy, chemotherapy, targeted therapy, and immunotherapy, are available. However, the prognosis remains poor owing to the high rates of tumor recurrence and distant metastasis (3, 4). Thus, there is an urgent need to identify novel therapeutic strategies for advanced HCC.

In recent years, pyroptosis has emerged as a prominent academic topic, particularly regarding its role in HCC (5–7). Pyroptosis is an inflammatory, caspase-1-dependent form of programmed cell death (8). Increasing evidence suggests that pyroptosis influences the progression of HCC by regulating tumor cell death. Studies analyzing RNA expression, prognosis, and immune infiltration of gasdermin D (GSDMD) in HCC indicate that GSDMD is both a prognostic marker and a potential therapeutic target for HCC (6, 9). Pyroptosis also impacts the tumor microenvironment in HCC, with cytokines such as IL-1β and IL-18 playing roles in regulating immune responses (10). This impact of pyroptosis highlights its potential as a target for liver cancer treatment. However, the mechanisms of pyroptosis in HCC remain underexplored.

Recent studies have focused on molecules that promote pyroptosis and regulate the activation of pyroptosis-related inflammasomes. Classical pyroptosis is associated with the NLRP3 inflammasome complex. Upon activation, the NLRP3 inflammasome activates caspase-1, which cleaves GSDMD and releases its pore-forming N-terminal domain. This cleaved GSDMD induces pyroptosis by forming pores in the plasma membrane (8, 11, 12). NADPH oxidase 4 (NOX4) is a member of the NADPH oxidase (NOX) family of proteins that constitutively produces H_2_O_2_ and reactive oxygen species (ROS). NOX4 plays a role in the regulation of HCC growth and signal transduction (13). The production of ROS enables the activation of NLRP3 inflammasomes, which in turn activates caspase-1 and leads to the release of IL-18, IL-1β, and lactate dehydrogenase (LDH) during canonical pyroptosis (14, 15). In recent years, more evidence has highlighted a close relationship between NOX4 and NLRP3, as well as their role in the mechanism of pyroptosis (16, 17). However, research on the mechanisms involving NOX4 in HCC remains relatively limited.

**Figure 1:**
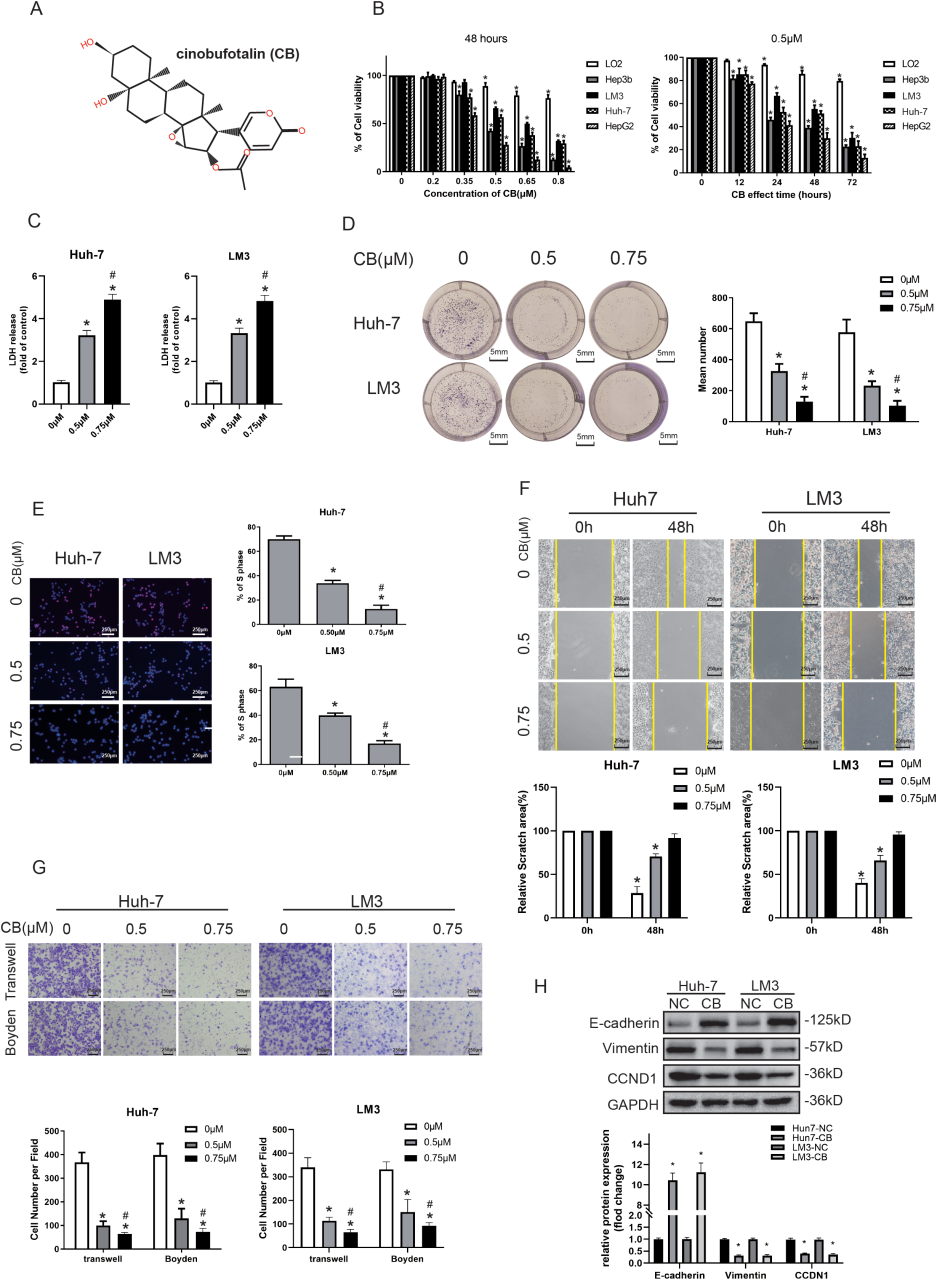
CB inhibited viability, proliferation, migration, and invasiveness of the HCC cells *in vitro*. **(A)** The chemical structure of CB. **(B)** Normal human hepatocyte cell line (LO2) and four different human hepatocellular carcinoma cell lines (Hep3b, Huh-7, LM3, and HepG2) were treated with various concentrations of CB for 48h or incubated with 0.5 μM of CB for different time (12, 24, 48, and 72 h). **(C)** Effects of CB on the LDH release in Huh-7 and LM3 cells. **(D, E)** Effects of CB on proliferation function of Huh-7 and LM3 were analyzed by colony-forming assay **(D)** and EdU incorporation assay **(E)**. **(F, G)** The migration and invasion ability of Huh-7 and LM3 with or without the stimulation of CB was inspected by wound-healing assays **(F)**, transwell migration assay **(G)**, and transwell invasion assay (Boyden Chamber assays) **(G)**, respectively. **(H)** Huh-7 and LM3 cells were incubated with CB (0.50 μM) for 48h. The expression of E-cadherin, Vimentin and CCND1 were assayed by western blot analysis. All experiments were performed in triplicate. Data are presented as mean ± SEM. *p < 0.05 versus 0mM of CB concentration treatment group or 0h group. #p<0.05 versus 0.5mM of CB concentration treatment group. NC, negative control; CB, Cinobufotalin; LDH, lactate dehydrogenase; CCND1, cyclin D1.

Cinobufotalin (CB, **Figure 1A**), a bufadienolide found in toad venom, is considered the main bioactive ingredient in CB injection, a traditional Chinese medicine (TCM) formulation (18). The antitumor activity of CB has been documented in several preclinical and clinical studies (19–22). Previous research has shown that pyroptosis can suppress tumor cell proliferation and metastasis, as well as alter the tumor microenvironment through the secretion of inflammatory cytokines (7, 12, 23). However, the antitumor effects of CB on pyroptosis, particularly in HCC, have not been reported. Therefore, in this study, we analyzed the effects of CB on HCC cells both *in vitro* and *in vivo*. Additionally, we investigated the mechanisms underlying the antitumor effects of CB in HCC, with a focus on its impact on pyroptosis through redox balance modulation and NLRP3 inflammasome activation.

## 2 Materials and methods

### 2.1 Ethics

The animal handling procedures in this study were in accordance with the Guide for the Care and Use of Laboratory Animals published by the National Institutes of Health and have been approved by the Branch for Medical Research and Clinical Technology Application, Ethics Committee of the First Affiliated Hospital of Fujian Medical University (MRCTA, ECFAH of FMU [2021]476). All animal experiments conducted were in full compliance with internationally accepted principles for the care and use of laboratory animals and conform to the Animal Research: Reporting of *In Vivo* Experiments (ARRIVE) guidelines.

### 2.2 Reagents

Cinobufagin (CB) was purchased from MCE (MedChemExpress, Shanghai, China). N‐acetylcysteine (NAC; superoxide inhibitor) was purchased from Sigma‐Aldrich (St. Louis, MO). VAS2870 (NOX4 inhibitor) and VX‐765 (caspase‐1 inhibitor) were purchased from SelleckChem (Houston, TX).

### 2.3 Xenograft model in nude mice

Four-five-week-old female BALB/c-nu/nu mice were purchased from the Shanghai Laboratory Animal Center (Shanghai, China) and housed in the animal facility under a sterile environment and a 12-h light/dark cycle. Isoflurane inhalation with a concentration of 3-5% was used as an anesthetic method. All mice were euthanized through cervical dislocation at the end of the experiment.

The subcutaneous xenograft mouse model was used to analyze the xenograft tumor growth. HCC cells (100 µl, 5×10^6^) were subcutaneously injected into the flanks of 4-5-week-old female mice. The mice were administered intraperitoneal injections of 8 mg/kg CB or equal volume of physiological saline once every day for 14 days and were sacrificed on the 15^th^ day. The xenograft tumors were harvested and analyzed. Tumor volume was determined using the following formula: (length × width^2^ × 0.50).

The pulmonary metastasis model was generated by injecting HCC cells (2.0×10^6^ cells per animal) through the tail vein of the nude mice. After one week, the mice were administered intraperitoneal injections of 8 mg/kg/day CB or equal volumes of physiological saline. All the mice were sacrificed on day 21. Lung samples were harvested and the status of lung metastasis were analyzed using hematoxylin and eosin (H&E) staining.

### 2.4 Immunohistochemical staining

Cancer specimens were incubated with the following primary antibody: Ki67 (ab16667; 1:200, Abcam, Cambridge, UK), NOX4(ab133303; 1:200, Abcam, Cambridge, UK), NLRP3 (ab270449, 1:200, Abcam, Cambridge, UK), Caspase-1 (22915-1-AP, 1:200, Proteintech, Rosemont, USA), and then incubated with streptavidin-biotin. Peroxidase conjugates were visualized with diaminobenzidine reagent.

### 2.5 Cell culture

All cell lines (Hep3b, Huh-7, LM3, HepG2 and LO2) were purchased from the Shanghai Zhong Qiao Xin Zhou Biotechnology Co. Ltd (Shanghai, China). The cells were cultured in the Dulbecco’s modified Eagle’s medium (DMEM, GIBCO, Life Technologies, Carlsbad, CA) supplemented with 10% fetal bovine serum (FBS, GIBCO, Life Technologies, Carlsbad, CA) in a humidified incubator maintained at 5% CO_2_ and 37°C.

### 2.6 Transient transfection with siRNAs

The small interfering RNAs (siRNAs) were designed and synthesized by RiboBio (Guangzhou, China). Huh 7 cells were transfected with the negative control (NC) and gene-specific siRNAs according to the manufacturer instructions. The transfection efficiency was 70%. The following siRNAs were used in this study: NOX4 (sense, 5′‐GGGCCAGAAUACUACUACATT‐3′; antisense, 5′‐UGUAGUAGUAUUCUGGCCCTT‐3′); NLRP (sense, 5′‐CCGCAUGAGCUUCGUCAAATT‐3′; antisense, 5′‐UUUGACGAAGCUCAUGCGGTT‐3′); Scrambled siRNA (5′-GATCCCCTTTCGTCCATCTCCA-3′ and 5′-AGCTTAAAAAT TTCGTCCATCG -3′).

### 2.7 Hydrogen peroxide assay

The hydrogen peroxide (H_2_O_2_) concentrations were estimated using the Hydrogen Peroxide Assay Kit (Beyotime, Shanghai, China) according to the manufacturer’s instructions. Briefly, the cells were lysed by incubation in the lysis buffer and centrifuged at 15,000×g for 5 min. The supernatants were collected and used for the assay. Equal volume of the supernatants were incubated with 100 μl of the reaction mixture at room temperature for 30 min. The optical density (OD) was measured at a wavelength of 560 nm. The concentration of H_2_O_2_ in the samples was calculated using the standard H_2_O_2_ calibration curve.

### 2.8 Estimation of intracellular ROS levels

ROS levels in the HCC cells were estimated using 2’,7’-dichlorofluorescein diacetate (DCFH_2_-DA; Molecular Probes, Life Technologies, NY, USA), a ROS-sensitive membrane-permeable fluorescent probe. Briefly, the cells after treatments were incubated with 10 μM DCFH_2_-DA at 37°C for 30 min in the dark. Then, the fluorescence of DCF-DA was quantified at an excitation wavelength of 480 nm and an emission wavelength of 525 nm using the SpectraMax M5/M5e multi-well fluorescence scanner (Molecular Devices, USA).

### 2.9 Lactate dehydrogenase assay

LDH in the culture medium was estimated using the LDH cytotoxicity assay kit (Nantong, Jiangsu, China). The assay was performed by incubating aliquots of the cell culture media from different groups of cells after treatments with the kit reagents at room temperature for 30 mins in a new microplate according to the manufacturer’s instructions. After stopping the reactions, LDH activity was determined by analyzing the OD value at 490 nm in a spectrophotometer.

### 2.10 Immunofluorescence

HCC cells (1 × 10^4^ cells/well) were seeded on glass coverslips in a 48-well plate and incubated overnight. Then, the cells were subjected to different treatments for different time points. The cells were fixed by incubation with 4% paraformaldehyde for 20 min at room temperature. Then, the cells were permeabilized with 0.1% Triton X-100 in PBS for 10 min and blocked with 5% BSA in TBS containing 0.05% Tween. Subsequently, the cells on the glass coverslips were incubated overnight at 4°C with primary antibodies against NLRP3 (ab270449, 1:50, Abcam, Cambridge, UK), ASC (ab283684, 1:100, Abcam, Cambridge, UK) and caspase-1 (22915-1-AP, 1:50, Proteintech, Rosemont, USA). After washing three times with the 1X TBS buffer, the coverslips were incubated with the following secondary antibodies: Cy3-conjugated anti-sheep Ab (red, 1:100; Beyotime, Shanghai, China), fluorescein isothiocyanate-conjugated anti-rabbit Ab (green, 1:100; Beyotime, Shanghai, China), and Alexa Fluor 405-conjugated anti-rabbit Ab(purple, 1:100; Beyotime, Shanghai, China). The nuclei was counterstained with 4′,6-diamidino-2-phenylindole (DAPI) (blue, 1:100; Beyotime, Shanghai, China). The fluorescent signals were visualized and photographed using an OLYMPUS FV10i-W confocal microscope (Olympus, Tokyo, Japan).

### 2.11 Western blotting

Total protein lysates were prepared from the cells and quantified using the BCA protein assay. Equal amounts of protein samples were separated on the SDS-PAGE and transferred onto PVDF membranes. Then, the membranes were blocked with 5% skimmed milk at room temperature for 1 h. Subsequently, the membranes were incubated overnight at 4°C with the following. The primary antibodies were E-cadherin(ab231303; 1:1000, Abcam, Cambridge, UK), Vimentin(ab92547; 1:1000, Abcam, Cambridge, UK), CCND1(ab1663; 1:200, Abcam, Cambridge, UK), NOX4(ab133303; 1:1000, Abcam, Cambridge, UK), NLRP3 (ab270449, 1:1000, Abcam, Cambridge, UK), ASC (ab283684, 1:1000, Abcam, Cambridge, UK), Caspase-1 (22915-1-AP, 1:2000, Proteintech, Rosemont, USA), IL-1β (ab234437, 1:1000, Abcam, Cambridge, UK), GSDMD (ab215203, 1:1000, Abcam, Cambridge, UK) and GAPDH (60004-1-Ig;1:7500, Proteintech, Rosemont, USA). The secondary antibodies were donkey anti‐mouse, donkey anti‐goat, and goat anti‐rabbit (1:15000; LI-COR Bioscience, Lincoln, NE). Immunoblot detection was performed using chemiluminescence with an Odysseys Infrared Imaging System.

### 2.12 MTT assay for cell viability

The effects of CB on the viability and proliferation of HCC cells (Huh 7 and LM3) was estimated using the 3-(4,5-dimethylthiazol-2-yl)-2,5-diphenyltetrazolium bromide (MTT) assays. For the cell viability assay, the cells were seeded into 96-well plates at a density of 5 × 10^3^ cells/well and were allowed to adhere by incubating overnight. Then, the cells were treated for various time points. For the proliferation assay, the cells were seeded in 96-well plates at a density of 1 × 10^3^ cells/well and treated with different concentrations of CB for 48 h or cultured for different lengths of time with the same concentration of CB. In both cases, after the treatments, 20 μl of MTT (5 mg/ml in PBS) was added into each well and the plates were incubated for another 4 h to allow formation of the formazan crystals. Then, the formazan crystals were dissolved with 200 μl of dimethyl sulfoxide (DMSO) (Sigma Aldrich, USA). The optical density (OD) value of each well was measured at a wavelength of 490 nm using the Synergy HT microplate reader (Bio-Tek).

### 2.13 Colony formation assay

HCC cells (Huh 7 and LM3) were trypsinized with 0.25% trypsin and single cell suspensions were prepared. Then, cells were seeded into 6-well plates at a density of 800 cells/well for 14 days. Subsequently, the cells were washed twice with PBS and stained with 0.1% crystal violet solution for 15 min at room temperature. Then, the colonies (≥50 cells per colony) were counted under a light microscope.

### 2.14 EdU incorporation assay

EdU incorporation assay was performed using the Cell-light EdU Apollo488 *In Vitro* Imaging Kit (RiboBio, Guangzhou, China) according to the manufacturer’s protocol. Briefly, HCC cells (Huh 7 and LM3) treated with or without CB were incubated with 10 μM EdU for 2 h. Then, the cells were fixed with 4% paraformaldehyde and permeabilized with 0.3% Triton X-100. The nuclei were stained with 5 μg/ml DAPI for 10 min. The number of Edu-positive cells were counted in five random fields under a fluorescent microscope.

### 2.15 Wound healing assay

Briefly, HCC cells (Huh 7 and LM3) treated with different concentrations of CB for 48 h were seeded overnight into 6-well plates (2×10^6^ cells/well) and grown until 100% confluency was achieved. Then, the monolayers of cells were wounded across the center of the well using a 200 μl pipette tip. The cells were washed twice to remove the detached cells and cell debris. Subsequently, the cells were incubated for 48 h in the maintenance medium (DMEM with 2% FBS). Subsequently, wound closure or cell migration was assessed by capturing images using a light microscope. The relative wound closure was calculated as a percentage of the occupied area in the gap relative to the initial scratch area.

### 2.16 Transwell migration and invasion assays

The transwell migration assay and transwell invasion assay were conducted with a transwell chamber(Corning, NY, USA). HCC cells were first incubated with different concentrations of CB for 48 h. In the migration assay, 1 × 10^5^ cells suspended in 100 μl serum-free DMEM were seeded in the upper compartment of the chamber and 600 μl DMEM with 10% FBS were added to the lower compartment of the chamber. The HCC cells were incubated for 16 h at 37°C. Subsequently, the migrating/invasive cells were fixed with methanol, stained with the Giemsa stain (Jiancheng, Jiangsu, China), and photographed under a light microscope. The total number of migrating or invasive cells were counted for each group. For the invasion assay, the upper compartment was precoated with 100 μl of Matrigel. All other processes were the same as for the transwell migration assay.

### 2.17 Enzyme-linked immunosorbent assay

The concentrations of IL-1β and IL-18 in the culture supernatants of HCC cells with or without CB treatment were estimated using the ELISA Kits (Life Technologies, Carlsbad, CA) according to the manufacturer’s instructions.

### 2.18 Serum measurements

Alanine aminotransferase (ALT), aspartate aminotransferase (AST) and creatinine (CR) were measured in serumusing biochemical kits (Nanjing Jiancheng Bioengineering Institute, Nanjing, China) following the manufacturer’s instructions.

### 2.19 Statistical analysis

The data were expressed as means ± standard deviation (S.D.). The differences between 2 groups were analyzed using the two-tailed Student’s t-test. The differences between multiple groups were analyzed using ANOVA. Statistical analyses were performed using the SPSS 22.0 software (SPSS Inc., Chicago, Illinois, USA). P < 0.05 was considered as statistically significant.

## 3 Results

### 3.1 CB treatment inhibits the viability, proliferation, migration, and invasiveness of HCC cells *in vitro*

We first analyzed the differential effects of CB on HCC cell lines compared with a normal hepatocyte cell line. The viability of CB-treated HCC cells (Hep3b, Huh-7, LM3, and HepG2) decreased in a concentration-dependent manner (0–0.8 μM) (**Figure 1B**). The 50% inhibitory concentration (IC_50_) for CB was 0.48 ± 0.04 μM, 0.64 ± 0.02 μM, 0.56 ± 0.03 μM, and 0.37 ± 0.01 μM for the Hep3b, LM3, Huh-7, and HepG2 cell lines (**Supplementary Figure S1**), respectively. Additionally, higher concentrations of CB reduced the viability of the normal human hepatocyte cell line, LO_2_, with an IC_50_ value of 1.49 ± 0.04 μM, which was significantly higher than those observed for the HCC cells (**Supplementary Figure S1**). Additionally, we observed a time-dependent (0–72 h) decrease in the viability of HCC cells treated with 0.5 μM CB, while LO_2_ cells maintained significantly higher viability (**Figure 1B**). This indicates that CB is less cytotoxic to LO_2_ cells compared with the HCC cell lines. Moreover, compared with the control group (untreated cells), CB-treated Huh7 and LM3 cells showed a dose-dependent increase in LDH release into the medium (**Figure 1C**). We also analyzed the effects of CB on the proliferation and colony formation ability of HCC cells. CB treatment significantly reduced both colony formation (**Figure 1D**) and EdU incorporation (**Figure 1E**) in HCC cells. These results demonstrate that CB treatment reduces the viability and proliferation of HCC cells *in vitro*.

The higher migration and invasiveness of HCC cells are associated with the poor prognosis of HCC tumors (3). Therefore, we investigated the effects of CB on the migration and invasiveness of HCC cells (Huh-7 and LM3) *in vitro*. HCC cells were incubated with different concentrations of CB (0, 0.5, and 0.75 μM) for 48 h, and their migration and invasion were analyzed using wound healing and transwell assays. The wound healing assay showed that CB treatment significantly decreased the migration of HCC cells compared with the untreated control groups *in vitro* (**Figure 1F**). The transwell migration assay, conducted without Matrigel pre-coating, showed that CB treatment significantly reduced the migration of HCC cells (**Figure 1G**). Similarly, the transwell invasion assay, conducted with Matrigel pre-coating, demonstrated that CB treatment significantly decreased the invasiveness of HCC cells (**Figure 1G**). These results indicate that CB effectively inhibits both the migration and invasiveness of HCC cells *in vitro*.

In previous studies, we found that CB regulates HCC cells mainly by inhibiting epithelial-mesenchymal transition (EMT) signaling and cell cycle regulatory proteins (24). In Huh-7 and LM3 cells, CB treatment led to the downregulation of EMT signals, specifically characterized by the upregulation of the E-cadherin protein and the downregulation of the vimentin protein. Additionally, the cell cycle regulatory protein, CCND1, was also downregulated in response to CB treatment (**Figure 1H**). These findings suggest that the ability of CB to regulate cell proliferation and migration may be attributed to its inhibition of EMT signaling and cell cycle proteins, although the specific regulatory mechanisms require further investigation.

### 3.2 CB inhibits proliferation and metastasis of xenografted HCC cells in nude mice *in vivo*

**Figure 2:**
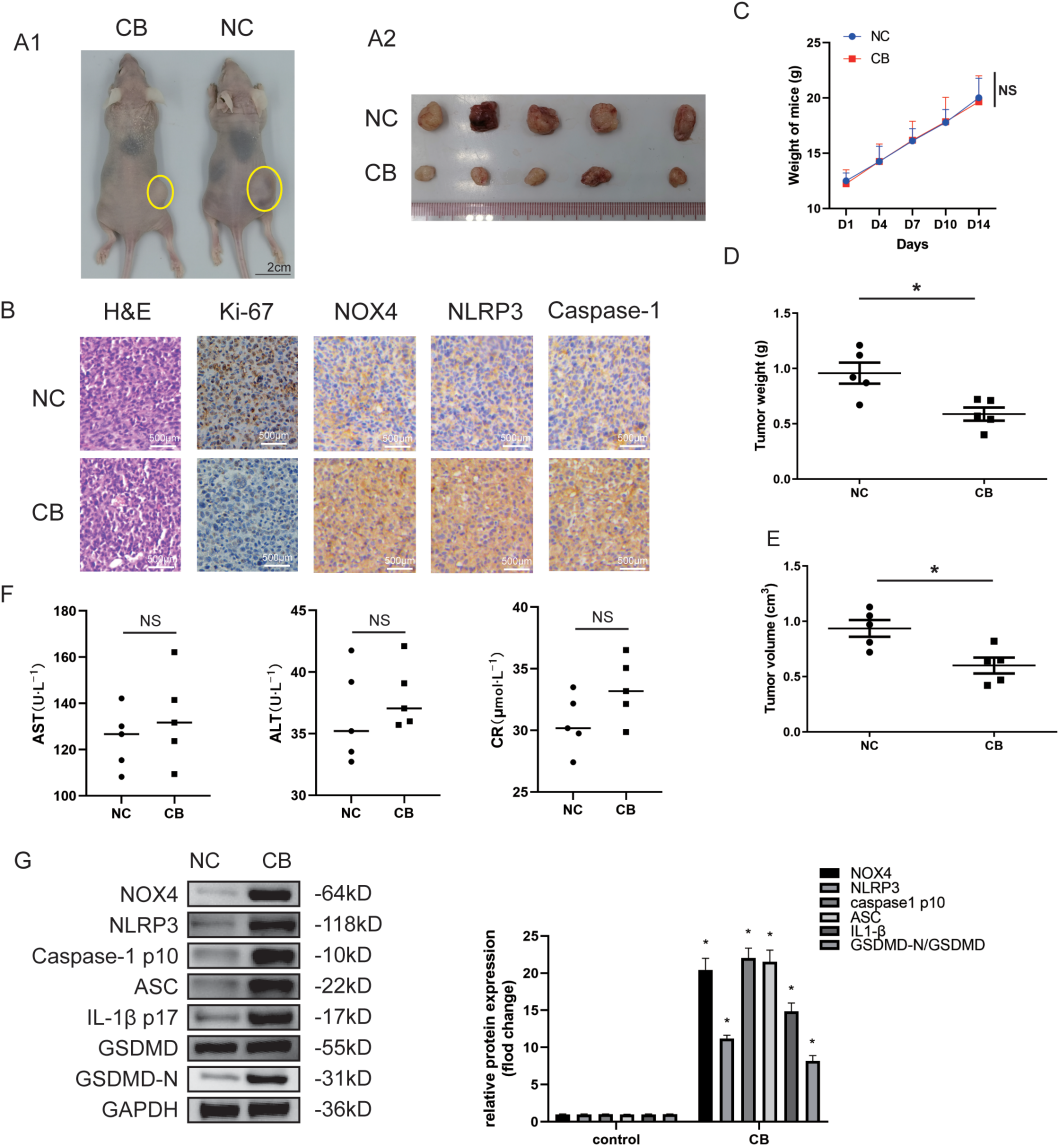
CB inhibited proliferation of the xenografted HCC cells in nude mice *in vivo*. **(A1)** Subcutaneous injection Huh7 cells with CB in nude mice to observe the effect of CB on tumor growth. **(A2)** The photo of tumors isolated from killed nude mice of the indicated groups. **(B)** Representative pictures of tumors sections stained with H&E and immunohistochemistry (Ki-67, NOX4, NLRP3, and Caspase-1). **(C)** The effect of CB on mice body weight. **(D)** The weight of the tumors. **(E)** The volume of the tumors. **(F)** The serum levels of ALT, AST, and CR. **(G)** The protein expressions of NOX4, NLRP3, Caspase-1 (p10), ASC, IL-1β (p17), GSDMD and GSDMD-N in tumor tissues were detected by western blotting. All experiments were performed in triplicate. Data are presented as mean ± SEM. *p < 0.05 versus NC group. The number of biological replicates for each experiment:5. NC, negative control; CB, Cinobufotalin; NLRP3, NOD‐like receptor family pyrin domain containing 3; ASC, Apoptosis-associated speck-like protein; IL‐1β, interleukin‐1β; GSDMD, Gasdermin D; NADPH, nicotinamide adenine dinucleotide phosphate; NOX4, NADPH oxidase 4; GAPDH, glyceraldehyde-3-phosphate dehydrogenase; HCC, Hepatocellular carcinoma; ALT, alanine aminotransferase; AST, aspartate aminotransferase; CR, creatinine; NS, no significance.

Subsequently, we assessed whether CB suppressed tumor formation from engrafted Huh-7 and LM3 cells in nude mice. The xenograft tumors in the CB group were significantly smaller compared with those in the negative control (NC) group (**Figure 2A** and **Supplementary Figure S2A**). Additionally, tumor weights (**Figure 2D** and **Supplementary Figure S2B**) and volumes (**Figure 2E** and **Supplementary Figure S2C**) were markedly reduced in the CB group relative to the NC group. Histological analysis also revealed decreased Ki67 staining in the xenograft tumors from the CB group compared with those from the NC group (**Figure 2B**). These results suggest that CB suppressed the proliferation of Huh-7 cells *in vivo*. Notably, no significant differences were observed in body weight (**Figure 2C**) or serum levels of AST (aspartate aminotransferase), ALT (alanine aminotransferase), and CR (creatinine) (**Figure 2F**) between the CB treatment group and the NC group, indicating that CB has minimal adverse effects *in vivo*.

**Figure 3:**
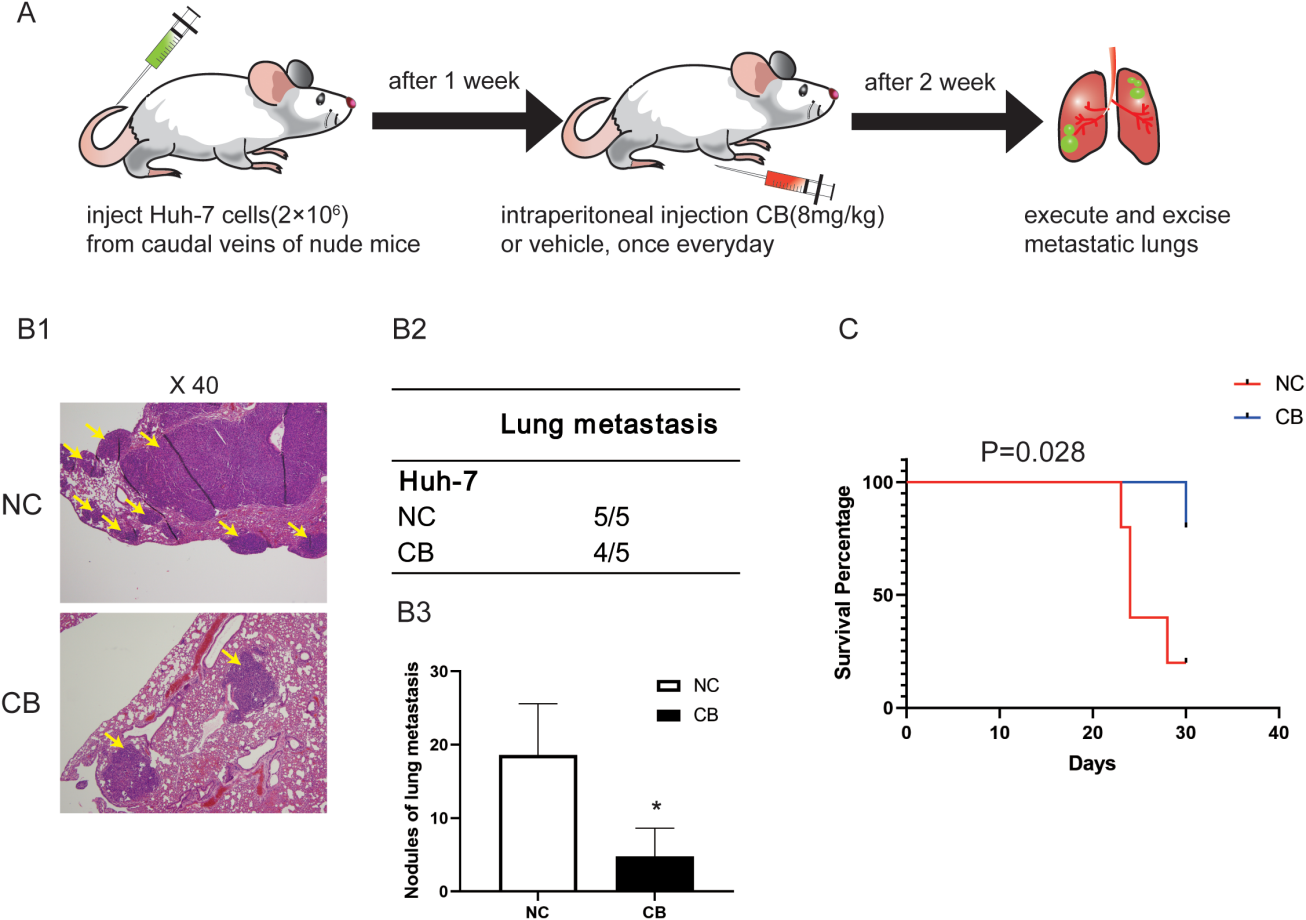
CB inhibited metastasis of the xenografted HCC cells in nude mice *in vivo*. **(A)** The pulmonary metastasis model was adopted to evaluate the effect of CB on metastasis in Huh-7 cells **(B1)** Representative pictures of lung metastatic nodules with H&E staining. **(B2)** The number of mice with pulmonary metastasis was calculated in each group. **(B3)** Statistics of pulmonary metastatic nodules. **(C)** The survival curves of mice. Data are presented as mean ± SEM. *p < 0.05 versus NC group. The number of biological replicates for each experiment:5. NC, negative control; CB, Cinobufotalin.

We then used a pulmonary metastasis model to assess the effects of CB on the migration and invasiveness of Huh-7 cells *in vivo* (**Figure 3A**). Lung metastasis was significantly reduced in the CB group compared with the NC group (**Figures 3B2-B3**). In contrast, the lungs of NC group mice were noticeably deformed and swollen, as confirmed by H&E-stained lung tissue sections (**Figure 3B1**). Additionally, CB treatment prolonged the survival time of mice with lung metastasis (**Figure 3C**). These data demonstrate that CB effectively inhibits the metastasis of Huh-7 cells *in vivo*.

Immunohistochemical staining revealed increased levels of NOX4, NLRP3, and caspase-1 (p10) proteins in the CB group (**Figure 1C**). Additionally, Western blot analysis showed that protein levels of NOX4, NLRP3, caspase-1 (p10), Apoptosis-associated speck-like protein (ASC), IL-1β (p17), and GSDMD-N were significantly increased in the CB treatment group compared with the NC group in xenograft tumors (**Figure 2G** and **Supplementary Figure S2D**). These results suggest that the inhibition of Huh-7 growth and progression by CB may be related to the activation of the NLRP3 inflammasome and NLRP3-related pyroptosis.

### 3.3 CB induces NLRP3/caspase-1/GSDMD-mediated pyroptosis

**Figure 4:**
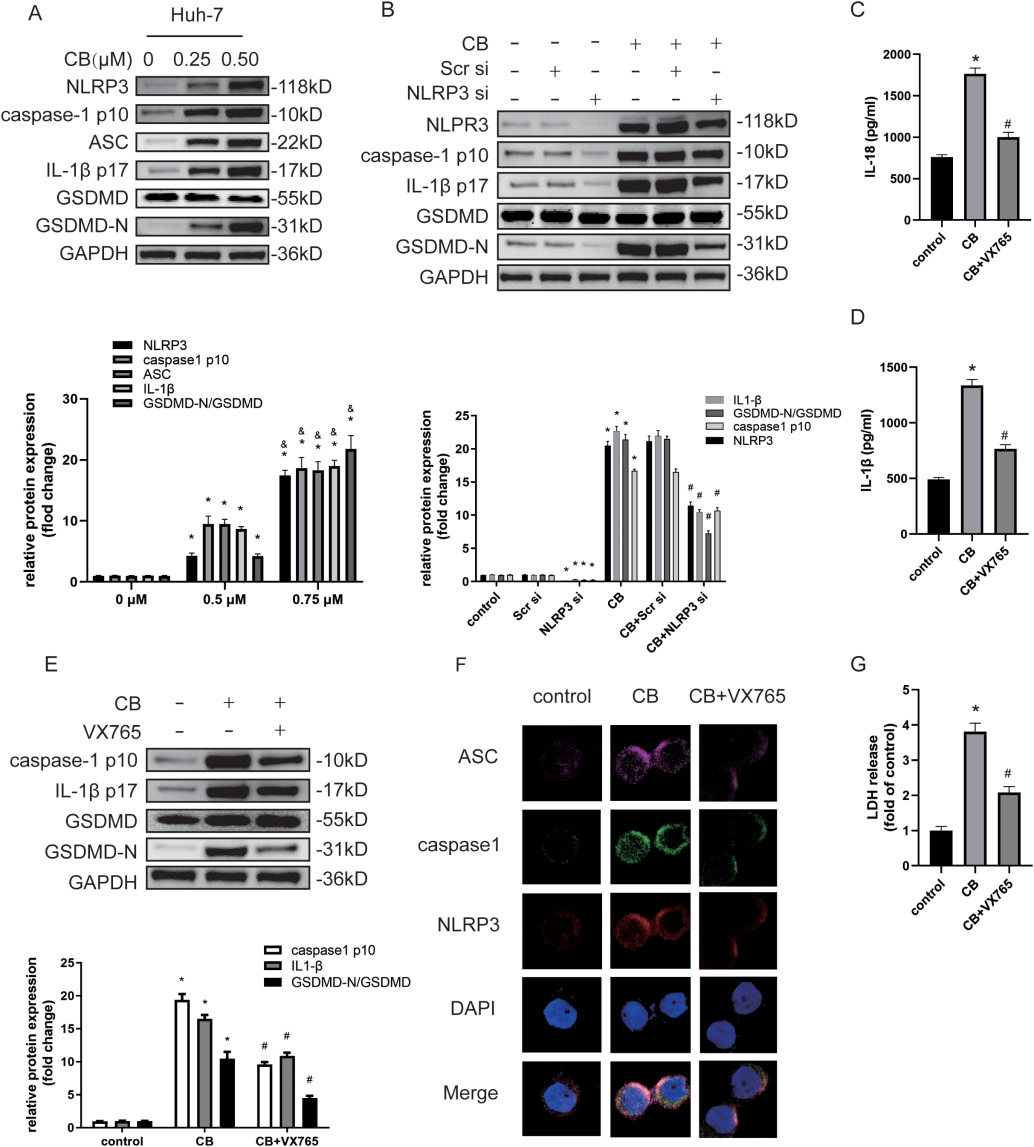
CB induced NLRP3/caspase-1/GSDMD-mediated pyroptosis. **(A)** Huh-7 cells were treated with CB (0.25, 0.50 μM) for 48h. The protein levels of NLRP3, Caspase-1 (p10), ASC, IL-1β (p17), GSDMD and GSDMD-N were assayed by western blot. **(B)** Huh-7 cells were pretreated with NLRP3 siRNA before stimulation with CB (0.50 μM) for 48h. The protein levels of NLRP3, Caspase-1 (p10), IL-1β (p17), GSDMD and GSDMD-N were assayed by western blot. **(C–G)** Huh-7 cells were pretreated with VX‐765 (10−4 M) for 1h before stimulation with CB (0.50 μM) for 48h. The culture supernatant was collected for IL-1β **(C)** and IL-18 **(D)** measurement by ELISA. The protein levels of Caspase-1 (p10), IL-1β (p17), GSDMD and GSDMD-N were measured by western blot analysis **(E)**. Huh-7 cells were stained for NLRP3 (red), ASC (purple), and caspase-1 (green) by immunofluorescence; Nuclei was stained with DAPI (blue) **(F)**. Original magnification: objective 60 ×, zoom: 2.0 ×. Effect of CB on the LDH release in Huh-7 cells **(G)**. All experiments were performed in triplicate. Data are presented as mean ± SEM. *p < 0.05 versus control group or 0mM of CB concentration treatment group; &p<0.5 versus 0.25mM of CB concentration treatment group; #p<0.50 versus CB group. CB, Cinobufotalin; NLRP3, NOD‐like receptor family pyrin domain containing 3; ASC, Apoptosis-associated speck-like protein; IL‐1β, interleukin‐1β; IL‐18, interleukin‐18; GSDMD, Gasdermin D; DAPI, 4′,6‐diamidino‐2‐phenylindole; siRNA, small interfering RNA; GAPDH, glyceraldehyde-3-phosphate dehydrogenase.

Previous studies have shown that the activity of the NLRP3 inflammasome regulates the proliferation and metastatic potential of HCC cells (14, 25). Therefore, we investigated whether CB treatment promotes the activation of the NLRP3 inflammasome and subsequent inflammasome-activated pyroptosis in Huh7 and LM3 cells. Western blot analysis demonstrated that CB treatment upregulated the expression levels of NLRP3, caspase-1 (p10), ASC, IL-1β (p17), and GSDMD-N proteins in a dose-dependent manner (**Figure 4A** and **Supplementary Figure S3A**). Furthermore, siRNA-mediated silencing of NLRP3 in Huh7 cells significantly inhibited the CB-induced upregulation of NLRP3, caspase-1 (p10), IL-1β (p17), and GSDMD-N proteins (**Figure 4B** and **Supplementary Figure S3B**). Additionally, treatment of Huh7 cells with VX765, a selective inhibitor of caspase-1, prevented the CB-induced upregulation of caspase-1 (p10), IL-1β (p17), and GSDMD-N proteins (**Figure 4E** and **Supplementary Figure S3E**). Subsequently, pretreatment with VX765 suppressed the secretion of IL-1β and IL-18 (**Figures 4C, D** and **Supplementary Figures S3C, D**) and the release of LDH (**Figure 4G** and **Supplementary Figure S3F**) in CB-stimulated Huh-7 cells. Triple immunofluorescence staining revealed co-localization of ASC (purple fluorescence), caspase-1 (green fluorescence), and NLRP3 (red fluorescence) in the cytoplasm of CB-treated Huh7 cells. Additionally, the expression levels of ASC, caspase-1, and NLRP3 were significantly increased in CB-treated Huh7 cells at 48 hours, but the assembly of the NLRP3 inflammasome complex was blocked by pretreatment with VX765 (**Figure 4F**). These results demonstrate that CB induces pyroptosis by activating the NLRP3 inflammasome in Huh7 and LM3 cells.

### 3.4 CB activates pyroptosis by inducing NOX‐derived ROS‐activated NLRP3 inflammasome

**Figure 5:**
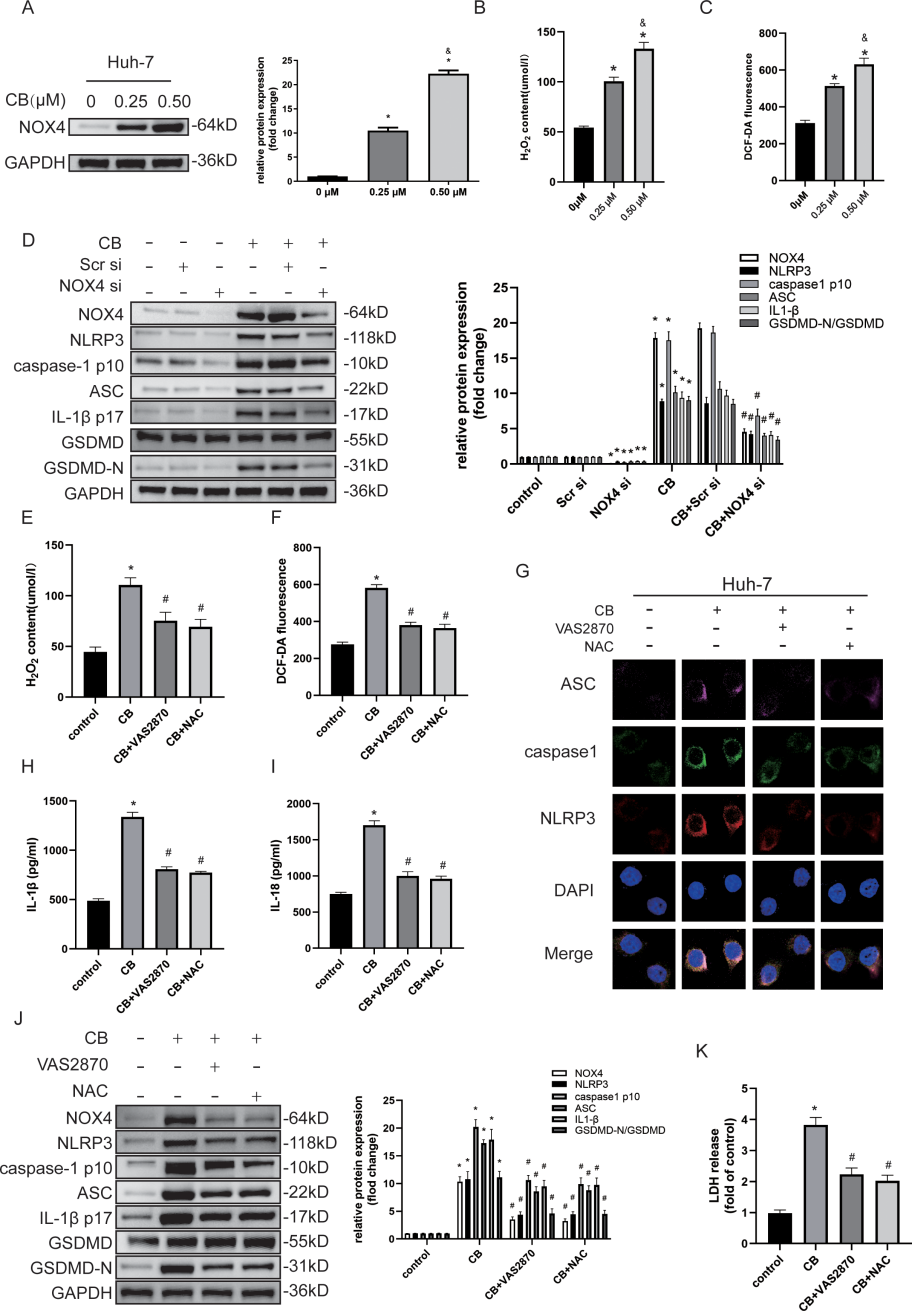
CB activated pyroptosis by inducing NOX‐derived ROS‐activated NLRP3 inflammasome. **(A)** Huh-7 cells were incubated with CB (0.25, 0.50 μM) for 48h. The expression of NOX4 was assayed by western blot analysis. **(B)** Levels of intracellular ROS in Huh-7 cells with or without indicated concentration of CB treatment was detected by the probe DCF-DA. **(C)** The concentration of H2O2 in Huh-7 cells with or without indicated concentration of CB treatment. **(D)** Huh-7 cells were pretransfected with NOX4 siRNA before stimulation with CB (0.50 μM) for 48h. The protein levels of NOX4, NLRP3, Caspase-1 (p10), ASC, IL-1β (p17), GSDMD and GSDMD-N were measured by Western blot. **(E–K)** Huh-7 cells were pretreated with VAS2870 (10−5 M) or NAC (10−3 M) before stimulation with CB (0.50 μM). **(E, F)** The concentration of H2O2 **(E)** and ROS **(F)** in Huh-7 cells. **(G)** Huh-7 cells were stained for NLRP3 (red), ASC (purple), and caspase-1 (green) by immunofluorescence; Nuclei was stained with DAPI (blue). Original magnification: objective 60 ×, zoom: 2.0 ×. **(H, I)** The culture supernatant was collected for IL-1β **(H)** and IL-18 **(I)** measurement by ELISA. **(J)** The protein levels of NOX4, NLRP3, Caspase-1 (p10), ASC, IL-1β (p17), GSDMD and GSDMD-N were measured by Western blot. **(K)** Effect of CB on the LDH release in Huh-7 cells. All experiments were performed in triplicate. Data are presented as mean ± SEM. *p < 0.05 versus control group or 0mM of CB concentration treatment group; &p<0.5 versus 0.25mM of CB concentration treatment group; #p<0.50 versus CB group. CB, Cinobufotalin; DAPI, 4′,6‐diamidino‐2‐phenylindole; NAC, N‐acetylcysteine; LDH, lactate dehydrogenase; NLRP3, NOD‐like receptor family pyrin domain containing 3; ASC, Apoptosis-associated speck-like protein; GAPDH, glyceraldehyde-3-phosphate dehydrogenase; IL‐1β, interleukin‐1β; IL‐18, interleukin‐18; GSDMD, Gasdermin D; NADPH, nicotinamide adenine dinucleotide phosphate; NOX4, NADPH oxidase 4; ROS, reactive oxygen species; siRNA, small interfering RNA.

Excessive intracellular ROS generation is associated with the activation of pyroptosis in HCC cells (15, 26). Evidence indicates that NOX4 plays a crucial role in ROS regulation in the liver (27, 28). Given that our data showed CB treatment inhibited the proliferation, migration, and invasiveness of Huh7 and LM3 cells, we investigated whether NLRP3 inflammasome-activated pyroptosis was triggered by elevated ROS levels. Western blot analysis demonstrated a dose-dependent increase in NOX4 protein levels in CB-treated Huh7 and LM3 cells (**Figure 5A** and **Supplementary Figure S4A**). Furthermore, CB-treated Huh7 and LM3 cells exhibited a dose-dependent increase in ROS and H_2_O_2_ levels (**Figures 5B, C** and **Supplementary Figures S4B, C**). However, both VAS2870 (a NOX4 inhibitor) and NAC (a ROS scavenger) reduced H_2_O_2_ and ROS levels in CB-treated Huh7 and LM3 cells (**Figures 5E, F** and **Supplementary Figures S4E, F**). Additionally, NOX4 silencing significantly inhibited the CB-induced upregulation of NLRP3, caspase-1 (p10), ASC, IL-1β (p17), and GSDMD-N protein levels in Huh7 and LM3 cells (**Figure 5D** and **Supplementary Figure S4D**). These findings suggest that NOX4 promotes the activation of the NLRP3 inflammasome and pyroptosis in CB-treated Huh7 and LM3 cells. Triple immunofluorescence staining showed that pretreatment with VAS2870 or NAC significantly inhibited the CB-induced assembly of the NLRP3 inflammasome complex (**Figure 5G**). Additionally, pretreatment with VAS2870 or NAC significantly reduced the CB-induced secretion of IL-1β and IL-18 (**Figures 5H, I** and **Supplementary Figures S4G, H**) and the release of LDH (**Figure 5K** and **Supplementary Figure S4J**) in Huh7 and LM3 cells. Furthermore, pretreatment with NAC or VAS2870 suppressed the CB-induced upregulation of NOX4, NLRP3, caspase-1 (p10), ASC, IL-1β (p17), and GSDMD-N protein levels in Huh7 and LM3 cells (**Figure 5J** and **Supplementary Figure S4I**). These data confirm that pyroptosis in CB-treated HCC cells is promoted by NOX4/ROS-induced activation of the NLRP3 inflammasome.

## 4 Discussion

TCM has been employed for over 2000 years to prevent and treat various diseases. The bioactive compounds in several TCM preparations have been shown to significantly inhibit cancer-related processes, including proliferation, metastasis, angiogenesis, and multidrug resistance. They also enhance anti-cancer effects, such as improving immunity and chemotherapy outcomes (29, 30). Additionally, these compounds regulate key biological processes related to cancer cell survival, including autophagy, oxidative damage, apoptosis, and pyroptosis (31, 32). CB is a natural bioactive compound with potent antitumor activity (18, 19, 21, 22). However, the antitumor effects of CB in HCC and the underlying mechanisms remain unclear. In this study, we found that CB inhibited the proliferation, migration, and invasiveness of HCC cells both *in vitro* and *in vivo*. Furthermore, CB exhibited no significant toxicity at the effective concentration. At the same concentration and exposure time, CB caused only minimal toxicity to the normal human hepatocyte cell line LO_2_ compared with the four HCC cell lines. These findings highlight the potential safety and efficacy of CB for clinical treatment of HCC. Previous studies have demonstrated that CB is well absorbed and widely distributed throughout body tissues and fluids. Additionally, CB exhibits good metabolic stability, low cytotoxicity, and acceptable bioavailability (19). Consequently, CB is considered a safe and effective adjuvant therapy for HCC.

Activation of the NLRP3 inflammasome can have both oncogenic and tumor-suppressive effects in HCC (14, 25). The NLRP3 inflammasome is composed of three proteins: NLRP3, apoptosis-associated speck-like protein (ASC) (an adaptor protein), and pro-caspase-1 (an effector protein). Upon stimulation, NLRP3 oligomerizes and interacts with ASC, which recruits and activates caspase-1 through self-cleavage. Activated caspase-1 then cleaves the precursors of IL-1β, IL-18, and gasdermin D into their mature and active forms (33). The NLRP3 inflammasome is central to multiple signaling pathways and inflammatory cytokines (14, 25). On one hand, its activation mediates pyroptosis and apoptosis, which can inhibit the development and progression of malignancies, including HCC. On the other hand, NLRP3 inflammasome activation can lead to excessive production of inflammatory cytokines such as IL-1β and IL-18, significantly affecting the tumor microenvironment and potentially promoting HCC growth (14, 25, 33). Therefore, the effect of CB on HCC depends on whether oncogenic or tumor-suppressive mechanisms are mainly activated through the NLRP3 inflammasome. In this study, western blotting and triple immunofluorescence staining results showed that CB treatment activated the NLRP3 inflammasome in HCC cells, as evidenced by elevated levels of NLRP3, ASC, cleaved caspase-1, cleaved IL-1β, and cleaved IL-18. Furthermore, CB treatment induced the release of cleaved IL-1β and IL-18, but these effects were mitigated by NOX4-specific siRNA and VX765, a caspase-1 inhibitor. Previous reports have indicated that dysregulation of the NLRP3 inflammasome is associated with poor pathological differentiation, advanced clinical stages, and a worse prognosis in patients with HCC (14, 25). Our findings support these reports and suggest that CB promotes the activation of the NLRP3 inflammasome, which in turn suppresses the proliferation, migration, and invasiveness of HCC cells.

Pyroptosis has emerged as a promising regulatory pathway with significant potential for cancer therapy (34, 35). This form of inflammation-related programmed cell death is characterized by pore formation, cell swelling, rapid cell rupture, and the release of cytokines such as IL-1β and IL-18 (8). Pyroptosis has a dual role in cancer pathogenesis. On one hand, it suppresses tumor occurrence and development by promoting cancer cell death. On the other hand, the inflammatory mediators released during pyroptosis and other pyroptosis-related signaling pathways can contribute to tumor resistance against chemotherapeutic drugs (36, 37). According to theories of inflammation-cancer transformation and chronic inflammation-induced carcinogenesis, pyroptosis, as a mode of proinflammatory cell death, can create a microenvironment conducive to tumor cell growth (12). Previous studies on HCC treatment have mainly demonstrated the antitumor effects of CB through inhibition of EMT and induction of apoptosis (38, 39). In this study, we focus on elucidating the mechanism by which CB induces pyroptosis in HCC cells. We first observed a dose-dependent increase in LDH release from CB-treated LM3 and Huh-7 cells, leading us to hypothesize that CB treatment induces pyroptosis in these HCC cells. Meanwhile, these changes are accompanied by an upregulation of E-cadherin expression and a downregulation of vimentin and CCND1 expression. This suggests that CB stimulation inhibits EMT signaling and cyclin-dependent kinase (CDK) expression. Previous studies have indicated that alterations in EMT and CDK expression may be key pathways regulating cell phenotype during pyroptosis (40–43). Further mechanistic research revealed that CB treatment significantly upregulated GSDMD-N expression, along with increased levels of NLRP3, ASC, cleaved caspase-1, and cleaved IL-1β in HCC cells. Additionally, CB-induced pyroptosis in Huh-7 cells was suppressed by either NLRP3-specific siRNA or the caspase-1 inhibitor VX765. These findings suggest that CB induces NLRP3/caspase-1/GSDMD-mediated pyroptosis in HCC cells. Evasion of apoptosis is a hallmark of cancer cells and is associated with resistance to antitumor therapies (44, 45). Therefore, inducing pyroptosis is crucial for treating tumors resistant to apoptosis. Our data suggest that CB may serve as an effective adjunct therapy for overcoming chemoresistance in HCC tumors by inducing pyroptosis.

Oxidative stress, characterized by an imbalance between the production and accumulation of ROS, plays a crucial role in the regulation of HCC growth and progression (46). NOX4, a non-phagocytic homolog of the NOX family, continuously produces hydrogen peroxide (H_2_O_2_) and ROS, which contribute significantly to HCC development. The deletion of the NOX4 gene is associated with higher tumor grades and worse recurrence-free and overall survival rates (13, 47). Our results indicated that the levels of NOX4, ROS, and H_2_O_2_ increased in a dose-dependent manner in CB-treated HCC cells. Pretreatment with NOX4 siRNA, NAC (an ROS scavenger), and VAS2870 (an NOX4 inhibitor) significantly reduced the expression levels of NLRP3, cleaved caspase-1, ASC, cleaved IL-1β, and GSDMD-N in the CB-treated HCC cells. These findings highlight the critical role of NOX4-generated ROS in NLRP3 inflammasome-mediated pyroptosis of HCC cells. Thus, CB appears to induce pyroptosis by promoting NOX4-dependent ROS production and the subsequent activation of the NLRP3 inflammasome through ROS.

Previous studies have demonstrated that members of the gasdermin family are crucial effectors in the mechanism of pyroptosis (8, 48). They not only mediate programmed necrosis of cells but also play a role in regulating the tumor immune microenvironment, including the modulation of PD-1/PD-L1 expression levels and Treg cell activity (35, 49, 50). Recent research has highlighted the significant role of gasdermin E (GSDME) in regulating and recruiting T cells in liver hepatocellular carcinoma and lung adenocarcinoma (51). Furthermore, tumors expressing GSDME exhibit increased macrophage phagocytic activity and elevated numbers and functions of natural killer cells (NK cells) and CD8^+^ T lymphocytes (52). The expression levels of gasdermin C (GSDMC) during pyroptosis are closely associated with the nuclear translocation of PD-L1 (53). GSDMD plays an active role in both pyroptosis and apoptosis (44). Additionally, GSDMD can enhance the expression of interferons by inactivating the cyclic guanosine monophosphate-adenosine monophosphate synthase (cGAS) pathway and downregulating PD-L1 expression. Researchers have hypothesized that combining anti-PD-1 therapy with a GSDMD inhibitor might be more effective in suppressing tumor growth than single treatments alone, as it could enhance cancer immunity (54). In this study, we confirmed that CB promotes pyroptosis in HCC cells by activating the classical caspase-1/GSDMD pathway, which inhibits cell proliferation, migration, and invasion. CB has demonstrated safety and efficacy as a pyroptosis inducer. However, further investigation is needed to explore the effects of CB on immune effector cells in models with established host immune systems. Introducing HCC cells into the huHSC-NOG-EXL transgenic mouse model could be a valuable experimental approach. This would enable the exploration of the effect of CB on immune cells, such as T cells, B cells, and macrophages, as well as its impact on PD-1 and PD-L1 expression. Additionally, this approach would allow for the investigation of whether CB can reverse resistance to PD-1 inhibitors.

The study has several limitations. Specifically, the effects and safety of CB as a therapeutic agent for HCC were not evaluated in patients. Future research will focus on assessing the therapeutic effect of CB in patients with HCC.

**Figure 6:**
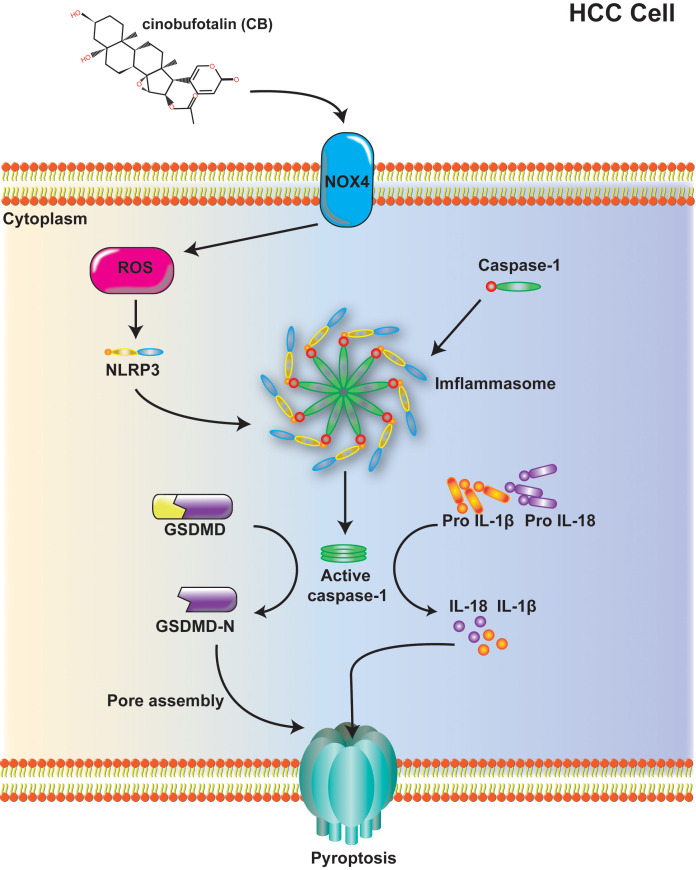
Mechanism diagram: Cinobufotalin inhibits proliferation, migration and invasion in hepatocellular carcinoma by triggering NOX4/NLRP3/GSDMD-dependent pyroptosis.

In conclusion, we have demonstrated for the first time that CB inhibits HCC by inducing NOX4/NLRP3/GSDMD-dependent pyroptosis both *in vivo* and *in vitro* (**Figure 6**). This study highlights a previously unrecognized role of CB in promoting pyroptotic cell death and establishes CB as a promising therapeutic agent for HCC.

## Data Availability

The original contributions presented in the study are included in the article/**Supplementary Material**. Further inquiries can be directed to the corresponding authors.

## Supplementary material

Supplementary Figure 1 CB IC50 values of the LO2 and HCC cells (Hep3b, LM3, Huh-7, HepG2). IC50, half maximal inhibitory concentration.

Supplementary Figure 2 CB inhibited proliferation of the xenografted HCC cells in nude mice *in vivo*. **(A)** Subcutaneous injection LM3 cells with CB in nude mice to observe the effect of CB on tumor growth. The photo of tumors isolated from killed nude mice of the indicated groups. **(B)** The weight of the tumors. **(C)** The volume of the tumors. **(D)** The protein expressions of NOX4, NLRP3, Caspase-1 (p10), ASC, IL‐1β (p17), GSDMD and GSDMD-N in tumor tissues were detected by western blotting. All experiments were performed in triplicate. Data are presented as mean ± SEM. *p < 0.05 versus NC group. The number of biological replicates for each experiment:5. NC, negative control; CB, Cinobufotalin; NLRP3, NOD‐like receptor family pyrin domain containing 3; ASC, Apoptosis-associated speck-like protein; IL‐1β, interleukin‐1β; GSDMD, Gasdermin D; NADPH, nicotinamide adenine dinucleotide phosphate; NOX4, NADPH oxidase 4; GAPDH, glyceraldehyde-3-phosphate dehydrogenase; HCC, Hepatocellular carcinoma, ALT, alanine aminotransferase, AST, aspartate aminotransferase, CR, creatinine.

Supplementary Figure 3 CB induced NLRP3/caspase-1/GSDMD-mediated pyroptosis. **(A)** LM3 cells were treated with CB (0.25, 0.50 μM) for 48h. The protein levels of NLRP3, Caspase-1 (p10), ASC, IL-1β (p17), GSDMD and GSDMD-N were assayed by western blot. **(B)** LM3 cells were pretreated with NLRP3 siRNA before stimulation with CB (0.50 μM) for 48h. The protein levels of NLRP3, Caspase-1 (p10), IL-1β (p17), GSDMD and GSDMD-N were assayed by western blot. **(C–F)** LM3 cells were pretreated with VX‐765 (10^−4^ M) for 1h before stimulation with CB (0.50 μM) for 48h. The culture supernatant was collected for IL-1β **(C)** and IL-18 **(D)** measurement by ELISA. The protein levels of Caspase-1 (p10), IL-1β (p17), and GSDMD-N were measured by western blot analysis **(E)**. Effect of CB on the LDH release in LM3 cells **(F)**. All experiments were performed in triplicate. Data are presented as mean ± SEM. ^*^p < 0.05 versus control group or 0μM of CB concentration treatment group; ^&^p<0.5 versus 0.25μM of CB concentration treatment group; ^#^p<0.50 versus CB group. CB, Cinobufotalin; NLRP3, NOD‐like receptor family pyrin domain containing 3; ASC, Apoptosis-associated speck-like protein; IL‐1β, interleukin‐1β; IL‐18, interleukin‐18; GSDMD, Gasdermin D; siRNA, small interfering RNA; GAPDH, glyceraldehyde-3-phosphate dehydrogenase.

Supplementary Figure 4 CB activated pyroptosis by inducing NOX‐derived ROS‐activated NLRP3 inflammasome. **(A)** LM3 cells were incubated with CB (0.25, 0.50 μM) for 48h. The expression of NOX4 was assayed by western blot analysis. **(B)** Levels of intracellular ROS in LM3 cells with or without indicated concentration of CB treatment was detected by the probe DCF-DA. **(C)** The concentration of H2O2 in LM3 cells with or without indicated concentration of CB treatment. **(D)** LM3 cells were pretransfected with NOX4 siRNA before stimulation with CB (0.50 μM) for 48h. The protein levels of NOX4, NLRP3, Caspase-1 (p10), ASC, IL-1β (p17), GSDMD and GSDMD-N were measured by Western blot. **(E–J)** LM3 cells were pretreated with VAS2870 (10−5 M) or NAC (10−3 M) before stimulation with CB (0.50 μM). **(E, F)** The concentration of H2O2 **(E)** and ROS **(F)** in LM3 cells. **(G, H)** The culture supernatant was collected for IL-1β **(G)** and IL-18 **(H)** measurement by ELISA. **(I)** The protein levels of NOX4, NLRP3, Caspase-1 (p10), ASC, IL-1β (p17), GSDMD and GSDMD-N were measured by Western blot. **(J)** Effect of CB on the LDH release in LM3 cells. All experiments were performed in triplicate. Data are presented as mean ± SEM. ^*^p < 0.05 versus control group or 0μM of CB concentration treatment group; ^&^p<0.5 versus 0.25μM of CB concentration treatment group; ^#^p<0.50 versus CB group. CB, Cinobufotalin; NAC, N‐acetylcysteine; LDH, lactate dehydrogenase; NLRP3, NOD‐like receptor family pyrin domain containing 3; ASC, Apoptosis-associated speck-like protein; GAPDH, glyceraldehyde-3-phosphate dehydrogenase; IL‐1β, interleukin‐1β; IL‐18, interleukin‐18; GSDMD, Gasdermin D; NADPH, nicotinamide adenine dinucleotide phosphate; NOX4, NADPH oxidase 4; ROS, reactive oxygen species; siRNA, small interfering RNA.
