# Supplementary figures and images for: Cinobufotalin inhibits proliferation, migration and invasion in hepatocellular carcinoma by triggering NOX4/NLRP3/GSDMD-dependent pyroptosis
Source: Front Oncol. 2024 Oct 16;14:1438306. doi: 10.3389/fonc.2024.1438306 (PMC11562471; doi:10.3389/fonc.2024.1438306)

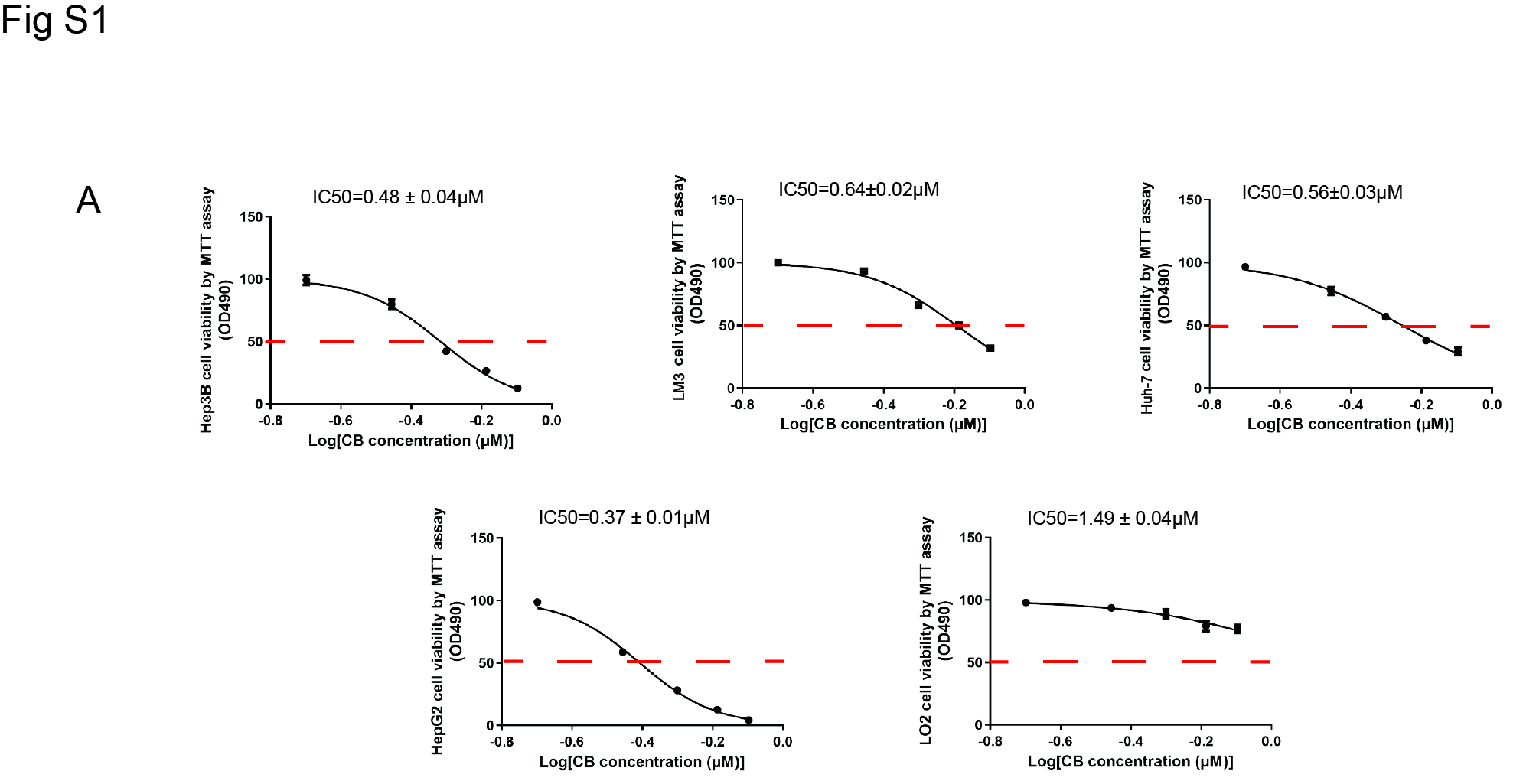

Supplement: Supplementary Figure 1 — CB IC50 values of the LO2 and HCC cells (Hep3b, LM3, Huh-7, HepG2). IC50, half maximal inhibitory concentration. [file Image1.tif]

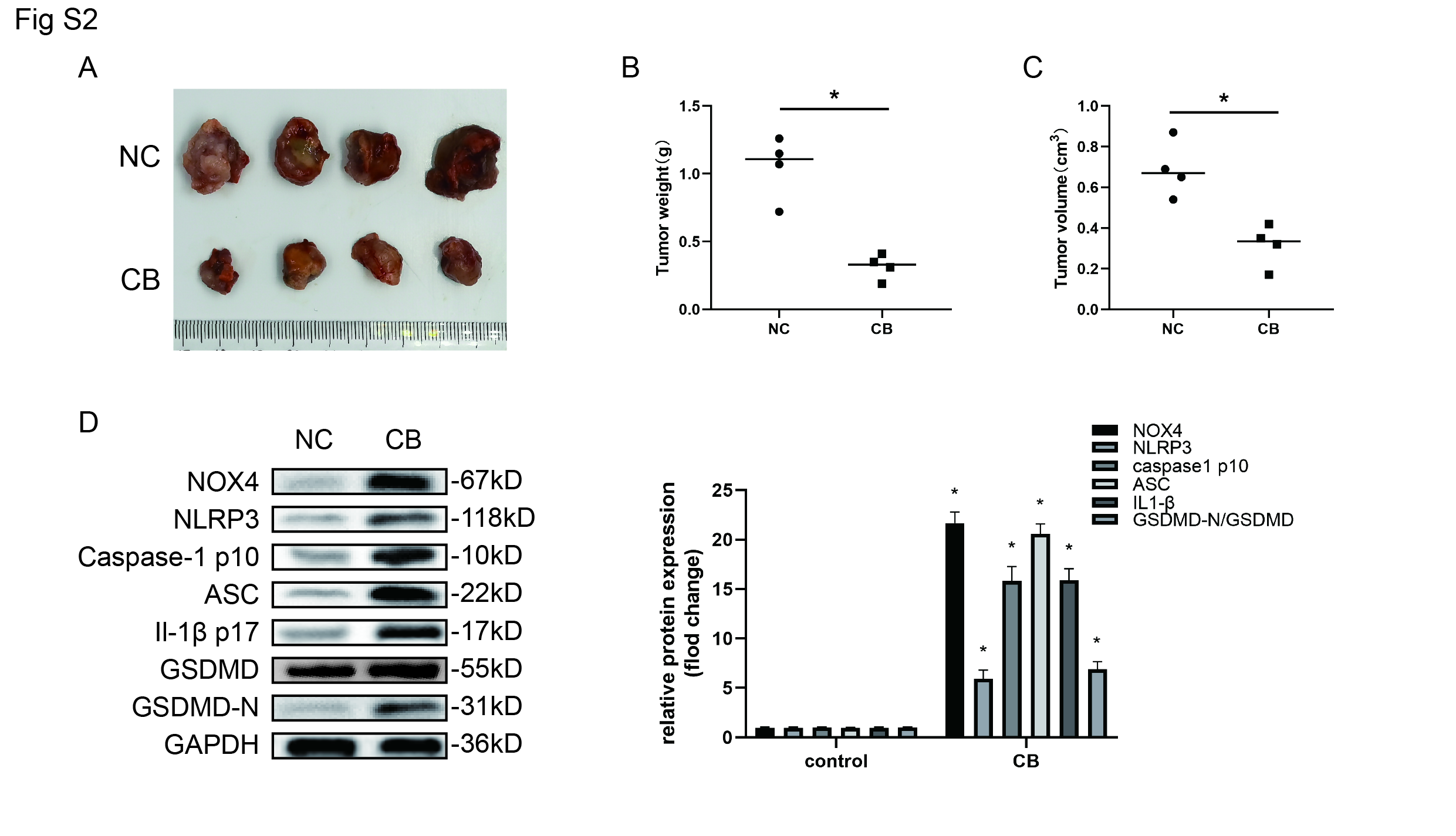

Supplement: Supplementary Figure 2 — CB inhibited proliferation of the xenografted HCC cells in nude mice in vivo. (A) Subcutaneous injection LM3 cells with CB in nude mice to observe the effect of CB on tumor growth. The photo of tumors isolated from killed nude mice of the indicated groups. (B) The weight of the tumors. (C) The volume of the tumors. (D) The protein expressions of NOX4, NLRP3, Caspase-1 (p10), ASC, IL‐1β (p17), GSDMD and GSDMD-N in tumor tissues were detected by western blotting. All experiments were performed in triplicate. Data are presented as mean ± SEM. *p < 0.05 versus NC group. The number of biological replicates for each experiment:5. NC, negative control; CB, Cinobufotalin; NLRP3, NOD‐like receptor family pyrin domain containing 3; ASC, Apoptosis-associated speck-like protein; IL‐1β, interleukin‐1β; GSDMD, Gasdermin D; NADPH, nicotinamide adenine dinucleotide phosphate; NOX4, NADPH oxidase 4; GAPDH, glyceraldehyde-3-phosphate dehydrogenase; HCC, Hepatocellular carcinoma, ALT, alanine aminotransferase, AST, aspartate aminotransferase, CR, creatinine. [file Image2.tif]

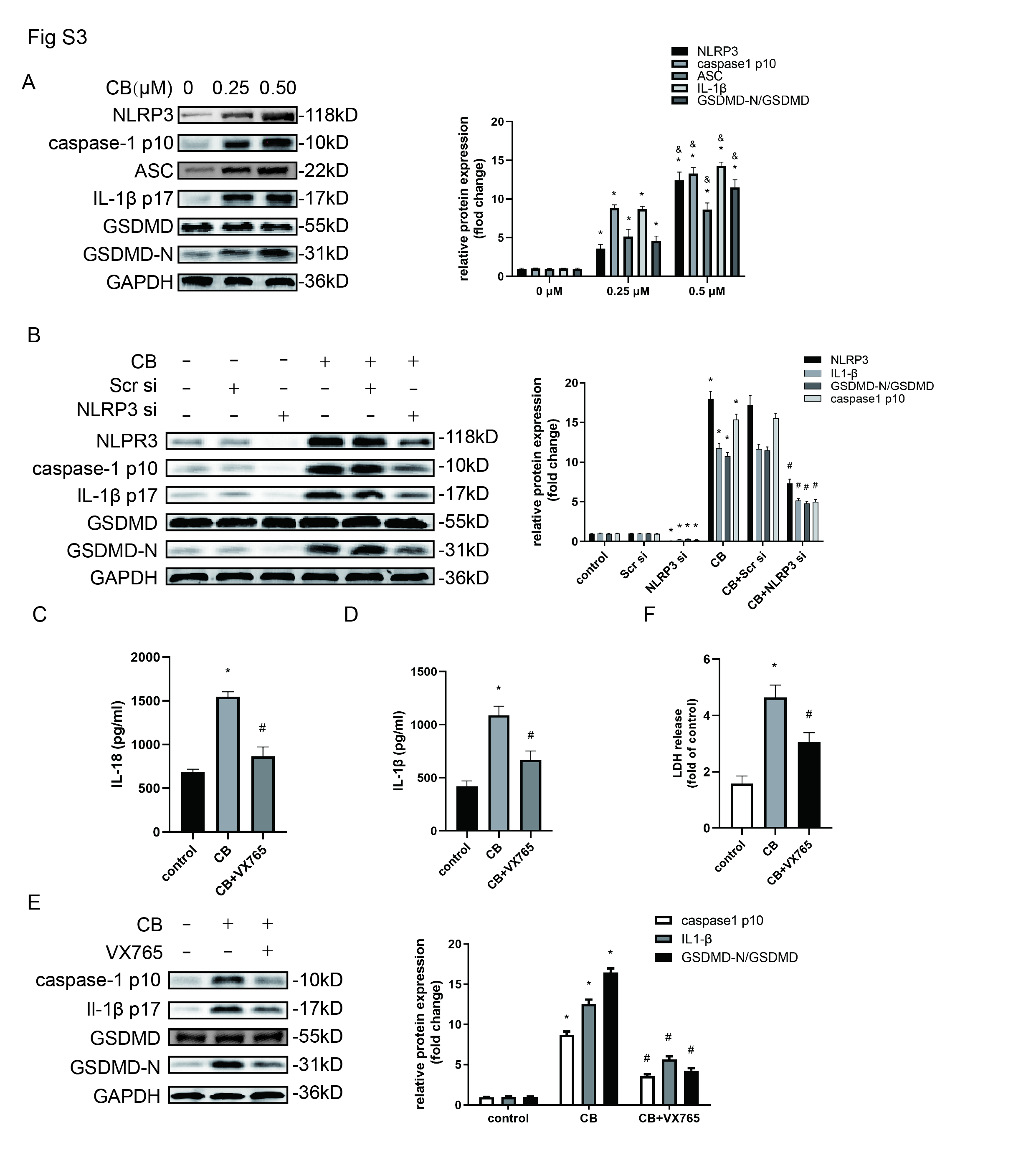

Supplement: Supplementary Figure 3 — CB induced NLRP3/caspase-1/GSDMD-mediated pyroptosis. (A) LM3 cells were treated with CB (0.25, 0.50 μM) for 48h. The protein levels of NLRP3, Caspase-1 (p10), ASC, IL-1β (p17), GSDMD and GSDMD-N were assayed by western blot. (B) LM3 cells were pretreated with NLRP3 siRNA before stimulation with CB (0.50 μM) for 48h. The protein levels of NLRP3, Caspase-1 (p10), IL-1β (p17), GSDMD and GSDMD-N were assayed by western blot. (C–F) LM3 cells were pretreated with VX‐765 (10−4 M) for 1h before stimulation with CB (0.50 μM) for 48h. The culture supernatant was collected for IL-1β (C) and IL-18 (D) measurement by ELISA. The protein levels of Caspase-1 (p10), IL-1β (p17), and GSDMD-N were measured by western blot analysis (E). Effect of CB on the LDH release in LM3 cells (F). All experiments were performed in triplicate. Data are presented as mean ± SEM. *p < 0.05 versus control group or 0μM of CB concentration treatment group; &p<0.5 versus 0.25μM of CB concentration treatment group; #p<0.50 versus CB group. CB, Cinobufotalin; NLRP3, NOD‐like receptor family pyrin domain containing 3; ASC, Apoptosis-associated speck-like protein; IL‐1β, interleukin‐1β; IL‐18, interleukin‐18; GSDMD, Gasdermin D; siRNA, small interfering RNA; GAPDH, glyceraldehyde-3-phosphate dehydrogenase. [file Image3.tif]

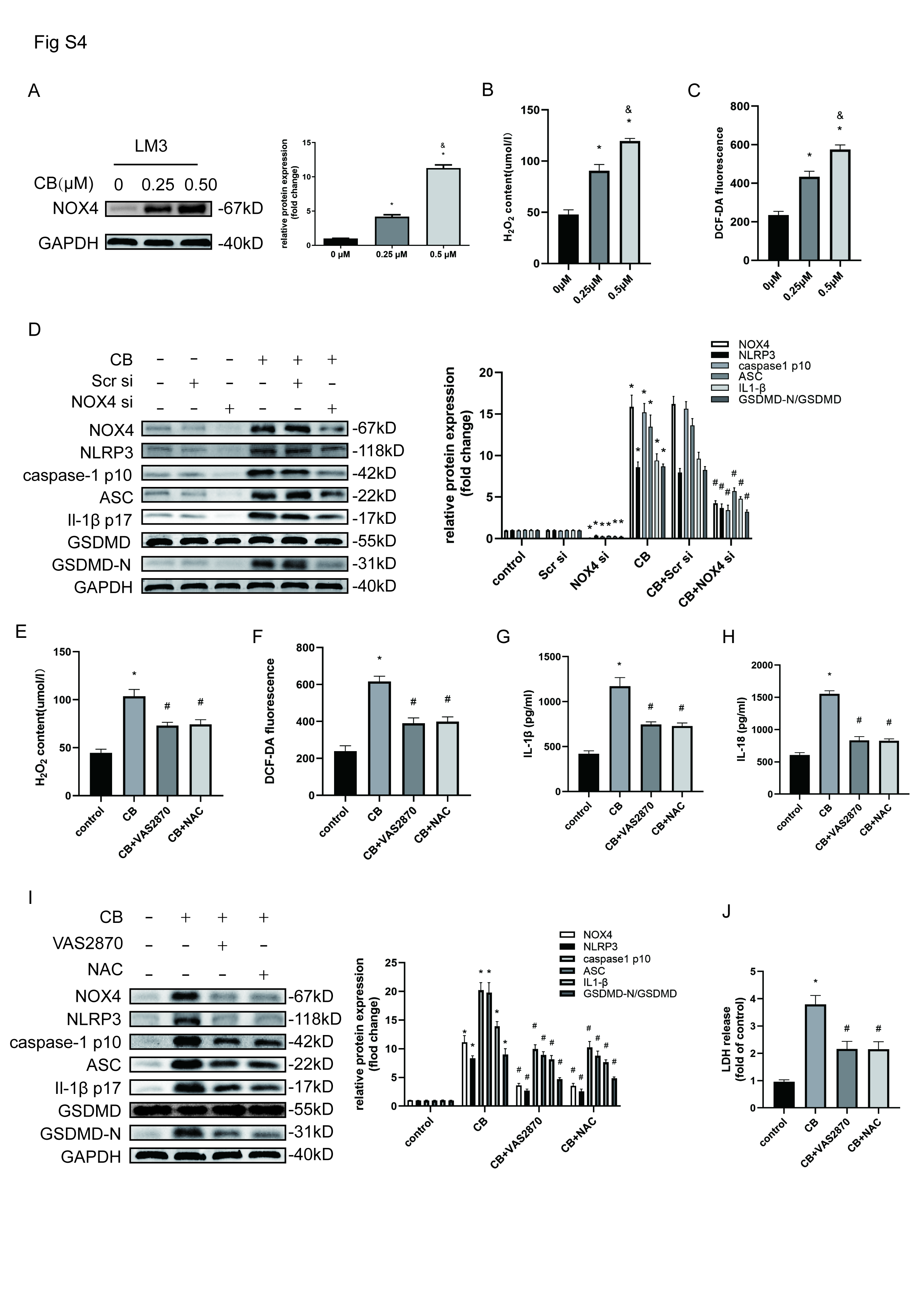

Supplement: Supplementary Figure 4 — CB activated pyroptosis by inducing NOX‐derived ROS‐activated NLRP3 inflammasome. (A) LM3 cells were incubated with CB (0.25, 0.50 μM) for 48h. The expression of NOX4 was assayed by western blot analysis. (B) Levels of intracellular ROS in LM3 cells with or without indicated concentration of CB treatment was detected by the probe DCF-DA. (C) The concentration of H2O2 in LM3 cells with or without indicated concentration of CB treatment. (D) LM3 cells were pretransfected with NOX4 siRNA before stimulation with CB (0.50 μM) for 48h. The protein levels of NOX4, NLRP3, Caspase-1 (p10), ASC, IL-1β (p17), GSDMD and GSDMD-N were measured by Western blot. (E–J) LM3 cells were pretreated with VAS2870 (10−5 M) or NAC (10−3 M) before stimulation with CB (0.50 μM). (E, F) The concentration of H2O2 (E) and ROS (F) in LM3 cells. (G, H) The culture supernatant was collected for IL-1β (G) and IL-18 (H) measurement by ELISA. (I) The protein levels of NOX4, NLRP3, Caspase-1 (p10), ASC, IL-1β (p17), GSDMD and GSDMD-N were measured by Western blot. (J) Effect of CB on the LDH release in LM3 cells. All experiments were performed in triplicate. Data are presented as mean ± SEM. *p < 0.05 versus control group or 0μM of CB concentration treatment group; &p<0.5 versus 0.25μM of CB concentration treatment group; #p<0.50 versus CB group. CB, Cinobufotalin; NAC, N‐acetylcysteine; LDH, lactate dehydrogenase; NLRP3, NOD‐like receptor family pyrin domain containing 3; ASC, Apoptosis-associated speck-like protein; GAPDH, glyceraldehyde-3-phosphate dehydrogenase; IL‐1β, interleukin‐1β; IL‐18, interleukin‐18; GSDMD, Gasdermin D; NADPH, nicotinamide adenine dinucleotide phosphate; NOX4, NADPH oxidase 4; ROS, reactive oxygen species; siRNA, small interfering RNA. [file Image4.tif]

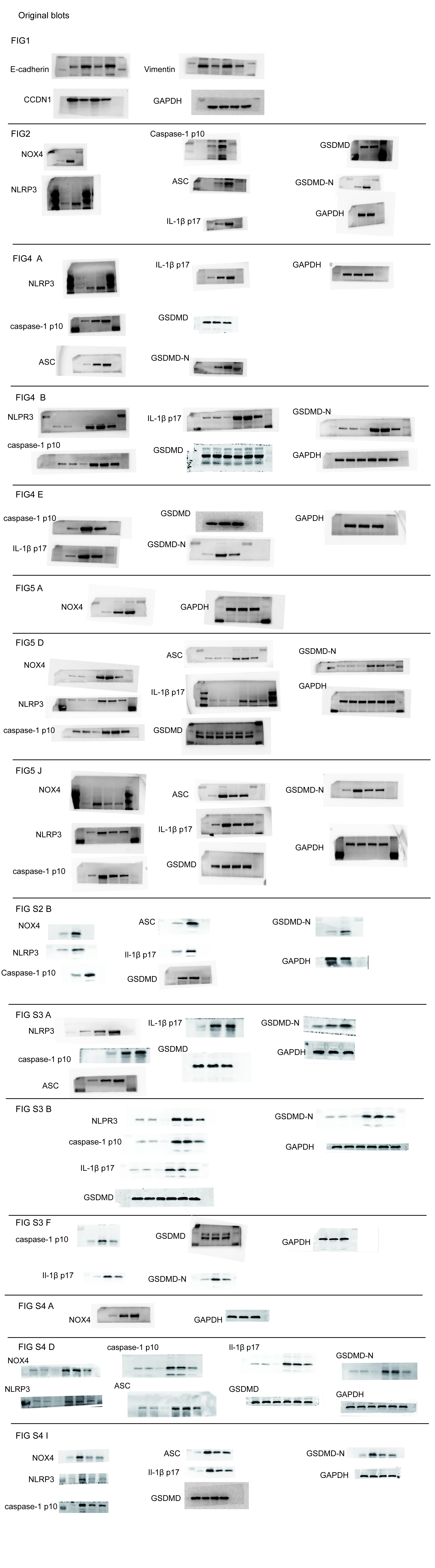

Supplement: Supplementary file 5 [file Image5.tif]
